# Aberrant activation of TGF-β/ROCK1 enhances stemness during prostatic stromal hyperplasia

**DOI:** 10.1186/s12964-024-01644-4

**Published:** 2024-05-06

**Authors:** Youyou Li, Jiaren Li, Liang Zhou, Zhenxing Wang, Ling Jin, Jia Cao, Hui Xie, Long Wang

**Affiliations:** 1 Department of Urology, The Third Xiangya Hospital, Central South University, Changsha, 410013 Hunan China; 2 Movement System Injury and Repair Research Center, Xiangya Hospital, Central South University, Changsha, 410008 Hunan China

**Keywords:** Prostatic hyperplasia, TGF-β, ROCK1, Prostatic stemness, PI3K/AKT

## Abstract

Benign prostatic hyperplasia (BPH) is a multifactorial disease in which abnormal growth factor activation and embryonic reawakening are considered important factors. Here we demonstrated that the aberrant activation of transforming growth factor β (TGF-β)/Rho kinase 1 (ROCK1) increased the stemness of BPH tissue by recruiting mesenchymal stem cells (MSCs), indicating the important role of embryonic reawakening in BPH. When TGF-β/ROCK1 is abnormally activated, MSCs are recruited and differentiate into fibroblasts/myofibroblasts, leading to prostate stromal hyperplasia. Further research showed that inhibition of ROCK1 activation suppressed MSC migration and their potential for stromal differentiation. Collectively, our findings suggest that abnormal activation of TGF-β/ROCK1 regulates stem cell lineage specificity, and the small molecule inhibitor GSK269962A could target ROCK1 and may be a potential treatment for BPH.

**Graphical Abstract:**

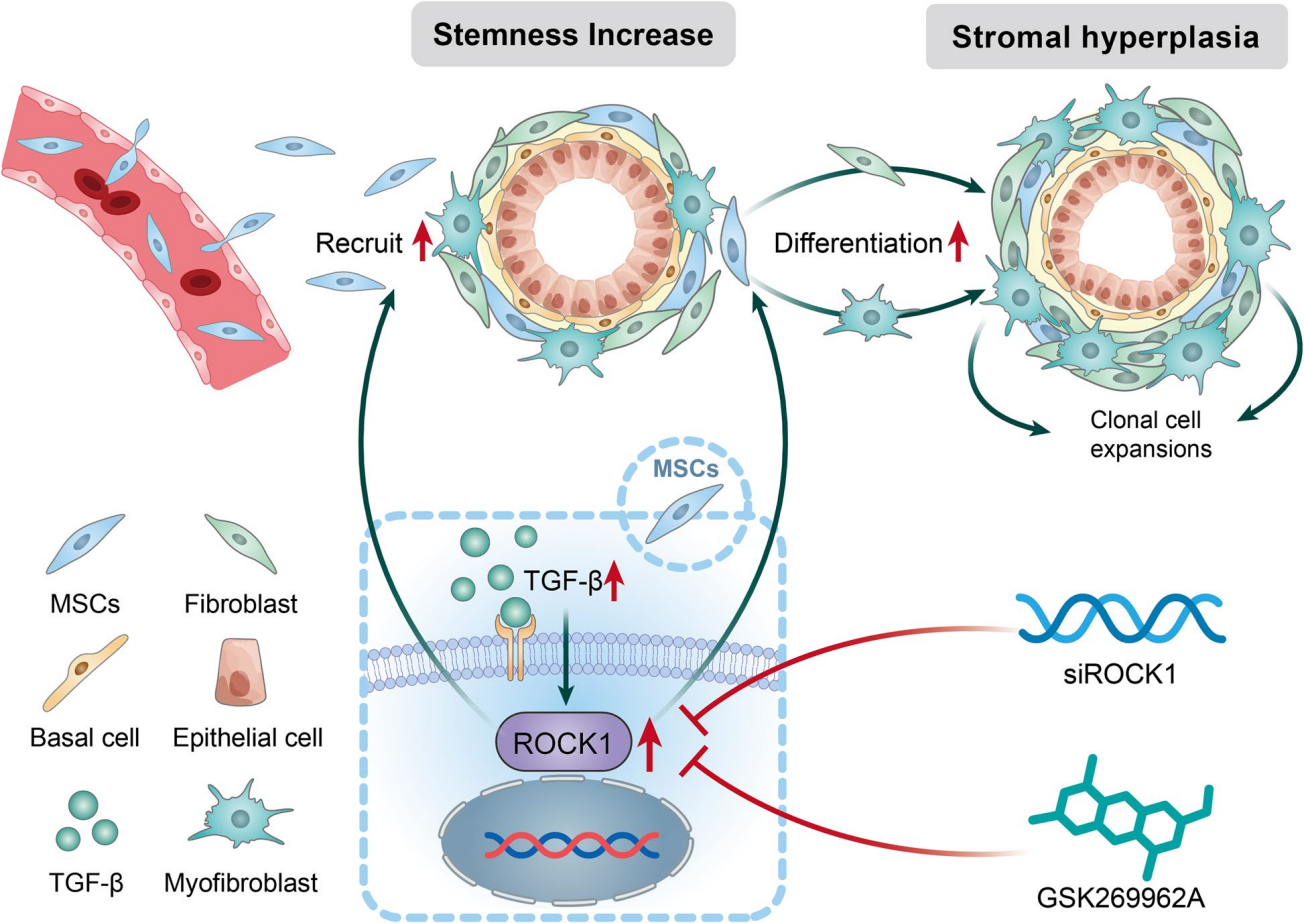

**Supplementary Information:**

The online version contains supplementary material available at 10.1186/s12964-024-01644-4.

## Introduction

Benign prostatic hyperplasia (BPH) is a common urological disease that affects older men worldwide [1, 2]. Due to the increase in the aging population worldwide, the incidence and prevalence of BPH have rapidly increased [3]. Approximately 50% of men over 60 years of age and 80% of men over 80 years of age are affected by BPH [4, 5]. Lower urinary tract symptoms (LUTS) related to BPH, recurrent urinary tract infections and acute urinary retention substantially impact men’s health and quality of life [6]. BPH is defined as the uncontrolled proliferation of connective tissue, smooth muscle, and glandular epithelium within the transitional zone of the prostate [7, 8], but the etiology of BPH is not fully understood and various pathways involving hormones, growth factors, inflammation, embryonic reawakening, and stem cells have been hypothesized to contribute to its pathogenesis [9].

During early development, the prostate undergoes a period of rapid growth and differentiation. BPH is believed to be a recapitulation of this early developmental process [9–11]. Histological analysis of BPH nodules revealed that they consist of stromal or mixed cell types [12]. In embryonic prostate development, prostatic rudiments emerge as buds from the urethral epithelium. These buds then expand into condensed clusters of mesenchymal fibroblasts in response to androgens [13]. BPH nodules undergo a comparable developmental process. Embryonic growth signals, such as TGF-β, recruit mesenchymal stem cells (MSCs) from the bone marrow, leading to the induction of new proliferative nodules within the mesenchyme [14].

Aberrant transforming growth factor β (TGF-β) signaling is involved in the pathogenesis of BPH, resulting in stromal proliferation, epithelial-mesenchymal transition (EMT), transdifferentiation, and extracellular matrix production [15–18]. Moreover, the expression of TGF-β receptor II protein in patients with BPH was shown to have a significant correlation with prostate volume and BPH inflammation [19]. TGF-β signal transduction involves both Smad-dependent and non-Smad-dependent pathways. Prostatic stromal cells positive for phosphorylated Smad2/3 (p-Smad2/3) show age-related expansion and accumulation, consistent with profibrotic TGF-β activation. Additionally, p-Smad2/3 both upregulates and downregulates mesenchymal and epithelial markers in prostate epithelial cells, facilitating EMT and contributing to the progression of BPH [20]. The Smad-independent pathways involved include the Rho kinase 1 (ROCK1) pathway, the phosphatidylinositol 3-kinase/protein kinase B pathway, the non-Smad JNK/p38 pathway, and the mitogen-activated protein kinase pathway [21, 22]. ROCK1 plays a regulatory role in various biological processes, such as actin cytoskeletal organization, cell adhesion, migration, proliferation, survival, and permeability [23, 24]. Notably, the TGF-β-activated ROCK1 signaling pathway is a determinant for the lineage commitment of MSCs in the abnormal deposition of collagen [25–27].

The existence of MSCs in BPH has been documented, highlighting their involvement in the disease [28, 29]. Our previous study identified a subset of MSCs activated by TGF-β in prostatic stromal hyperplasia [30]. The leptin receptor serves as a marker for prospective identification and in vivo fate mapping of bone marrow MSCs. LepR^+^ MSCs exhibit stemness properties, including self-renewal and the potential to differentiate into multiple cell types [31]. Stemness encompasses the intrinsic capacity of a cell to maintain its lineage, generate differentiated cells, and interact with its microenvironment to regulate quiescence, proliferation, and regeneration, thereby allowing the cell to give rise to all cell types in the adult organism [32]. However, the investigation of stemness within prostate tissue in BPH remains largely unexplored and warrants further scientific inquiry. Recently, TGF-β was shown to regulate the expression of stemness factors, suggesting that TGF-β may enhance stemness by recruiting MSCs, leading to the reawakening of the prostate stroma [33, 34].

In summary, this study investigated the relationship between TGF-β signaling, stromal hyperplasia, and stemness in BPH. Additionally, we explored the role of LepR^+^ MSCs and the ROCK1 signaling pathway in the pathogenesis of BPH, while also validating the potential of ROCK1 as a therapeutic target. Importantly, this study provides strong support for the theory of embryonic reawakening in BPH.

## Methods and materials

### Human specimens

A total of 35 BPH specimens were collected from patients with BPH who underwent transurethral prostate resection in the Department of Urology, the Third Xiangya Hospital of Central South University. As controls, 9 normal prostates were obtained from bladder tumor patients younger than 50 years who underwent radical cystoprostatectomy. The normal prostates were reviewed by three pathologists to confirm that they were normal prostate tissue without any evidence of cancer invasion. The stromal/epithelial ratio was approximately 2:1. This experiment complies with the Declaration of Helsinki and was performed under the oversight and regulations of the ethics committee of the Third Xiangya Hospital (No. 22239).

### Animal experiments

Transgenic animal experiments were carried out on 4- to 10-week-old mice. Trangenic Leprtm2(cre)Rck/J (Stock No. 008320) and Gt(ROSA26)Sor(tdTomato-WPRE) (Stock No. 007909) mice were purchased from Jackson Laboratories (California, USA). LepR-Cre mice were crossed with tdTomato transgenic mice to generate LepR-tdTomato mice. Mouse genotyping was performed under the guidance of the JAX genotyping protocols, and primer sequences are listed in Table S1. All mice were maintained under specific pathogen-free conditions with 12 h light/12 h dark. The treatment lasted for 4-weeks and involved administering the ROCK inhibitor GSK269962A (5 mg/kg, MCE) or the equivalent dose of solvent was injected intraperitoneally four times a week to male wild-type mice aged 8-weeks. Phenylephrine (PE) injection was used to initiate the treatment. The animal experiment was authorized by the ethics committee of Central South University (Reference No. 2021sydw0039).

### Parabiosis model

Parabiosis mouse models were constructed as previously described [35]. Nine six-week-old male LepR-tdTomato (LepR-cre;tdTomato^fl/−^) mice were randomly surgically conjoined to a wild-type male littermate (Strain matched). The successful establishment of shared blood circulation was proven by flow cytometry of peripheral blood. The littermates were injected subcutaneously with PE (10 mg/kg^−1^/day^−1^), while 3'-O-CH3 modified siROCK1 (Sangong, China) or the same mock as the control was injected into the tail vein for 4 weeks prior to sacrifice. Mice were anesthetized with tribromoethanol, and blood and prostate tissue were collected from the wild-type littermates for analysis.

### Hematoxylin and Eosin (HE) and Masson’s trichrome staining

For HE staining, paraffin-embedded tissues were dewaxed, rehydrated, HE stained, and dehydrated. Masson’s trichrome staining was performed according to standard procedures using the Trichrome Stain (Masson) kit (Sigma-Aldrich).

### Immunohistochemistry and immunofluorescence

Five-micrometer slices were cut from formalin-fixed, OCT or paraffin-embedded specimens. The standard immunochemistry procedure of was followed, and then, the sections were incubated with the corresponding primary antibody at 4℃ overnight, followed by a 1-h incubation with biotinylated secondary antibodies or fluorescent secondary antibodies at room temperature. Representative images from each stained slice were captured by Imager.M2 (Zeiss, Germany). Microscopic images were assessed using Image J (https://imagej.nih.gov/ij/) to calculate the integral optical density (IOD) and the area of each picture. The average optical density was defined as the integrated optical density divided by area.

### Wound healing and Transwell assays

A scratch wound healing assay was performed to examine the collective migration of LepR^+^ MSCs. After transfection of LepR^+^ cells with 100 nM siROCK1 (RiboBio, China) as described in the manual, the cells were added to a 6-well plate containing 2 ml of serum-free α-MEM with or without TGF-β (10 ng/ml, Sino Biological, China). After culture for 12 h, a scratch was made with a pipette tip. The width of the scratch gaps was recorded. The cells were cultured for 24 h. Then the width of the wound gaps was photographed. For Transwell migration assays, the siROCK1-transfected cells were counted and reseeded on the upper face of Transwell migration chambers (Costar 3422, Corning). Then, 500 μl of α-MEM with 10% FBS was added to the lower chamber. After culture for 24 h, the Transwell was removed and fixed with 0.4% paraformaldehyde. Then, 0.1% crystal violet was added for 15 min, and the cells were washed twice with PBS. The cells on the upper surface were wiped away. Cells attached to the lower surface were observed under a microscope (Nikon, Japan) and photographed.

### Flow cytometry

Mouse peripheral blood was collected using anticoagulation tubes, lysed by red blood lysis solution (Solarbio, # R1010) and resuspended by adding 0.5% BSA PBS buffer. Nonspecific antibody binding to the FC receptor was blocked by preincubating the cells with Trustain Fcx PLUS (Biolegend, #156,604, reactive to CD16/CD32), and samples were then stained with corresponding antibodies and analyzed by flow cytometry.

### RT‒qPCR

Total RNA was prepared by using TRIzol and reverse‐transcribed into cDNA with the NovoScript Plus All-In-One kit (Novoprotein, China). Real‐time quantitative PCR (RT–qPCR) was performed using the 2 × (Selleck, USA) and FTC 3000 Real‐time PCR system (Funglyn Biotech, CA), with GAPDH as a reference control. Primer information is detailed in Table S1.

### Western blot

Total protein was extracted from cell samples, and the concentration was determined using a BCA protein assay kit (Elabscience, China). Samples were separated by sodium dodecyl sulfate/polyacrylamide gel electrophoresis and transferred onto polyvinylidene fluoride membranes. Blots were incubated with primary antibodies followed by HRP‐conjugated secondary antibodies and developed with enhanced chemiluminescence (Advansta, USA). Antibody information is detailed in Table S2.

###  Isolation and culture of mouse tdTomato-labeled LepR + cells

8-week-old LepR-tdTomato mice were euthanized and the prostate ventral lobe was carefully harvested and dissected under a microscope, and then minced and digested with prewarmed collagenase (Gibico, #17,101,015) solution (Collagenase type II was diluted to a final concentration of 1 mg/mL in α-MEM.) for 3 h at 37 °C. The dissociated tissue chunks were further washed with PBS once and placed in 0.25% trypsin for 10 min at 37 °C, and digestion was terminated by adding 5% FBS (Biological Industries, Israel), followed by centrifugation at 300 g for 5 min. Subsequently, the dissociated cell suspension was filtered with a 70-mm nylon cell strainer. The dissociated prostate cells were stained for 15 min on ice with CD45. Cell sorting was performed on a FACS Aria II system (BD). The tdTomato^+^ CD45^–^ cells were sorted and cultured with α-MEM containing 1% penicillin/streptomycin (PS) sulfate (Thermo Scientific, USA), and 10% FBS at 37 °C in a 5% CO^2^ humidified incubator.

### Multilineage differentiation

Prostatic LepR^+^ cells were isolated as abovementioned. The cells were seeded onto a 48-well plate and cultured in growth medium (α-MEM with 10% FBS, 1% P.S) for 24 h. Then different induction media (Cyagen, China) were used for adipogenic, osteogenic, and fibrogenic differentiation. The medium was changed every two days. In vitro adipogenic and osteogenic differentiation of LepR^+^ MSCs was performed following previous methods [36]. The experimental medium for fibroblast differentiation consisted ofα-MEM growth medium plus 100 ng/mL recombinant human connective tissue growth factor (R&D Biovender, USA) and 50 μg /mL ascorbic acid (Sigma-Aldrich).

### Collection and analysis of related signatures

Twenty-six stemness gene sets were obtained from a web-based tool: StemChecker [37]. Six prostate stem cell-related gene sets were obtained from Yan et al. [38]. A total of 225 TGF-β related genes were identified from several common public databases, including "BIOCARTA_TGFB_PATHWAY", "KEGG_TGF_BETA_SIGNALING_PATHWAY" and "GO:0007179". The EMT signature, AR signature, and ER signature were obtained from MSigDB and included "HALLMARK EPITHELIAL MESENCHYMAL TRANSITION", "HALLMARK ANDROGEN RESPONSE", "HALLMARK ESTROGEN RESPONSE EARLY", and "HALLMARK ESTROGEN RESPONSE LATE". The relative scores of BPH samples were quantified by single-sample gene set enrichment analysis (ssGSEA) in the R package "gsva" [39]. Correlations were determined by Spearman’s correlation coefficient. Related signatures are detailed in Table S3.

### Prediction of the therapeutic response

The drug sensitivity prediction for each patient based on bulk RNA-seq data was performed using the "oncoPredict" R package. The "oncoPredict" team has updated and uploaded the datasets required for fitting the ridge regression model, which includes resources from the Genomics of Drug Sensitivity in Cancer [40].

### Molecular docking

The X-ray crystal structures of ROCK1 (PDB: 7JOU) were retrieved from the Protein Data Bank. The protonation state of all the compounds was set at pH = 7.4, and the compounds were expanded to 3D structures using Open Babel [41]. AutoDock Tools (ADT3) were applied to prepare and parametrize the receptor protein and ligands. The docking grid documents were generated by AutoGrid of sitemap, and AutoDock Vina (1.2.0) was used for docking simulation [42, 43]. The optimal pose was selected to analyze the interaction. Finally, the protein ligand interaction figure was generated by PyMOL. The ROCK1 protein is represented as a slate cartoon model, the ligand is shown as a cyan stick, and their binding sites are shown as magenta stick structures. Nonpolar hydrogen atoms are omitted. The hydrogen bonds, ionic interactions, and hydrophobic interactions are depicted as yellow, magenta and green dashed lines, respectively.

### Statistical analysis

The data are presented as mean ± SEM or SD. Paired individual values were compared using unpaired tests. A Wilcoxon signed-rank test was employed for stemness and estimated IC50 comparison. For multiple comparisons, analysis of variance (ANOVA) with Bonferroni’s post hoc test was conducted where applicable. GraphPad Prism 9 software was utilized for statistical analysis. Statistical significance was defined as *p* < 0.05.

## Results

### Stromal hyperplasia and hyperactivation of TGF-β are important features of BPH

**Fig. 1:**
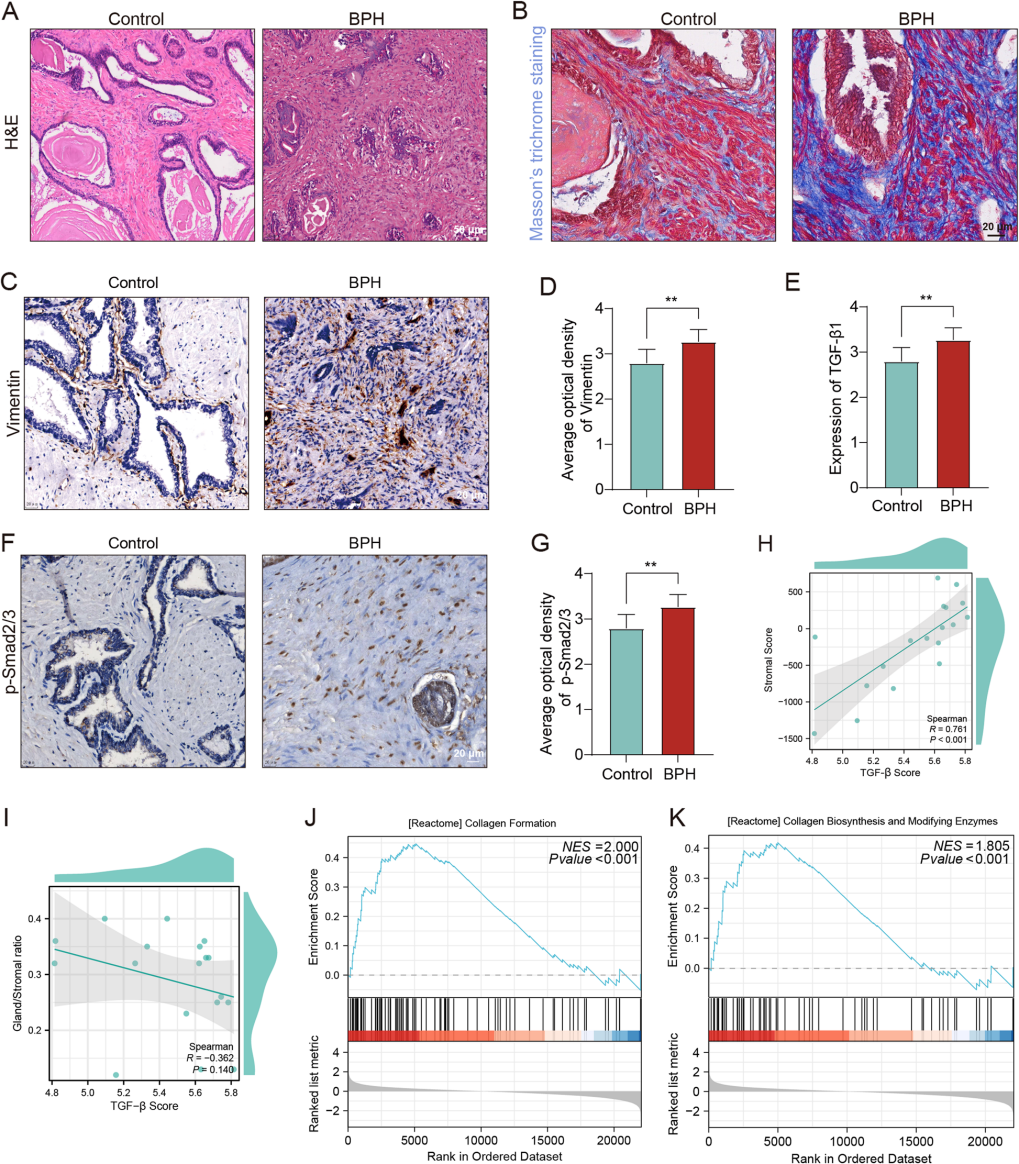
Expression of active TGF-β and stromal accumulation in prostatic hyperplasia tissue. **A** HE staining of human normal prostate(left) and BPH (right) tissues. Scale bars = 50 μm. **B** Representative images of Masson trichrome staining of collagen deposition (blue) in prostate tissues. Scale bars = 20 μm. **C-D** Expression level of vimentin protein (brown) in BPH and normal prostate tissues. The results were expressed as mean optical density. Scale bars = 20 μm. **E** Expression of TGF-β1 mRNA between BPH and normal prostate tissues in GSE132714. **F-G** Expression level of p-Smad2/3 protein (brown) in BPH and normal prostate tissues. The results were expressed as mean optical density. Scale bars = 20 μm. **H-I** The Spearman correlation between the TGF-β score, stromal score and gland/stromal ratio. **J-K** GSEA of stromal fibrosis gene sets between high and low TGF-β score groups. Data represent means ± SEMs (n = 10 or 5 per group). **p < 0.01

The progression of BPH is primarily triggered by increased stromal components and prostatic fibrosis. HE and Masson staining of BPH tissue revealed a notable increase in stromal components and collagen deposition (Fig. 1A-B). Moreover, vimentin expression was significantly elevated in the stroma of BPH tissue compared to normal prostate tissue (Fig. 1C-D). Aberrant activation of the TGF-β signaling pathway is a characteristic feature of the stromal microenvironment in BPH. This conclusion was validated through analysis of GEO datasets and immunohistochemistry, which demonstrated upregulation of TGF-β1 expression and downstream signaling in BPH (Fig. 1E-G). However, the precise biological role of TGF-β in the pathogenesis of BPH remains unclear.

Numerous studies have demonstrated that TGF-β promotes stromal cell proliferation and induces EMT in epithelial cells [15, 16, 44]. To further investigate the potential association among TGF-β scores, stromal scores, and the gland/stromal cell ratio, we performed correlation analysis. The results revealed a strong positive and statistically significant correlation between the TGF-β score and stromal score (R = 0.761, Fig. 1H), as well as a negative correlation with the gland/stromal cell ratio (R = -0.362, F ig. 1I). However, the latter correlation was not significant.

Collagen deposition is an important feature of prostatic stromal hyperplasia. Gene set enrichment analysis (GSEA) indicated enhancement of pathways commonly associated with collagen, such as collagen formation and collagen biosynthesis and modifying enzyme pathways, in the high TGF-β score group (Fig. 1J-K). These findings suggest that aberrant activation of TGF-β signaling and stromal hyperplasia are not only important features of BPH but also indicate a potential significant relationship between these factors. However, the underlying mechanism of this association is unknown.

Given the significant role of TGF-β responses in prostatic stromal hyperplasia, we established a PE-induced mouse model following established protocols [30]. As depicted in Figure S1A, the mouse prostate index [calculated as prostate weight (mg) divided by body weight (g)] exhibited a significant 1.2-fold increase in the group of mice receiving subcutaneous injections of PE. Histological examination of HE and Masson staining prostate sections from the PE-induced mice revealed evident stromal compartment hyperplasia and papillary accumulation of epithelial cells compared to those of the saline-treated mice after 4 weeks of injection (Fig. S1B-C). Consistent with expectations, there was a significant increase in the number of vimentin^+^ fibroblasts in the prostate stroma, and immunostaining for p-Smad2/3 revealed upregulated expression of p-Smad2/3 in both epithelial and stromal cells following continuous PE injection for 4 weeks (Fig. S1D-E).

### TGF-β in BPH patients associated with stemness

**Fig. 2:**
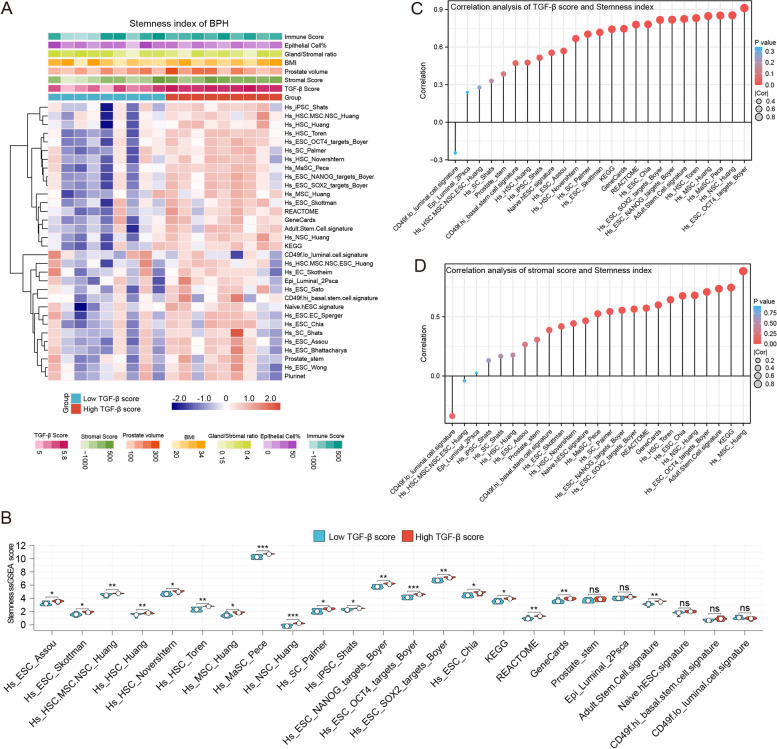
Stemness was correlated with different TGF-β scores in BPH patients. **A** Heatmap depicting the landscape of the ssGSEA stemness index in high or low TGF-β score groups. **B** Boxplot displaying the 26 ssGSEA stemness index between two groups by Wilcoxon rank sum test. **C** The correlation between the TGF-β score and stemness index. Each column represents a stemness gene set. **D** The correlation between stromal score and stemness index. Each column represents a stemness gene set. *p < 0.05, **p < 0.01, ***p < 0.001

Stromal reawakening is one of the most important theories in the pathogenesis of BPH [11]. Supporting evidence for this theory includes the presence of MSCs in human BPH tissue [28]. Hence, evaluation of the stemness of each BPH sample using the ssGSEA algorithm based on the stemness gene set is theoretically feasible. This investigation revealed that the group with the high TGF-β score exhibited a higher stemness index, which is consistent with the involvement of TGF-β superfamily signaling pathways in early developmental events such as embryonic patterning and cell fate determination. Most of the stemness indices showed significant differences between the high and low TGF-β groups (Fig. 2A). Interestingly, four prostatic stemness indices did not display significant differences between the two groups, indicating that stem cells originating from outside the prostate may play a crucial role in the pathogenesis of BPH rather than prostate stem cells (Fig. 2B).

Subsequently, we investigated the relationships between the stemness index and the TGF-β score and stromal score. The stemness index exhibited positive associations with both the TGF-β score and stromal score in GSE132714, further validating the strong correlation between the TGF-β score and stemness index in the GSE101486 dataset. (Fig. 2C-D, Fig. S2A). Notably, a positive association was primarily observed for the transition zone volume and one specific stemness index (Fig. S2B). Overall, these findings demonstrate a strong correlation between the abnormal activation of TGF-β, the stemness index, and the stromal score in BPH. Moreover, increased stemness may contribute to the benign volume enlargement observed in the transitional zone of the prostate.

###  Circulating LepR^+^ MSCs recruited to the reactive stroma acquire fibrotic phenotypes

**Fig. 3:**
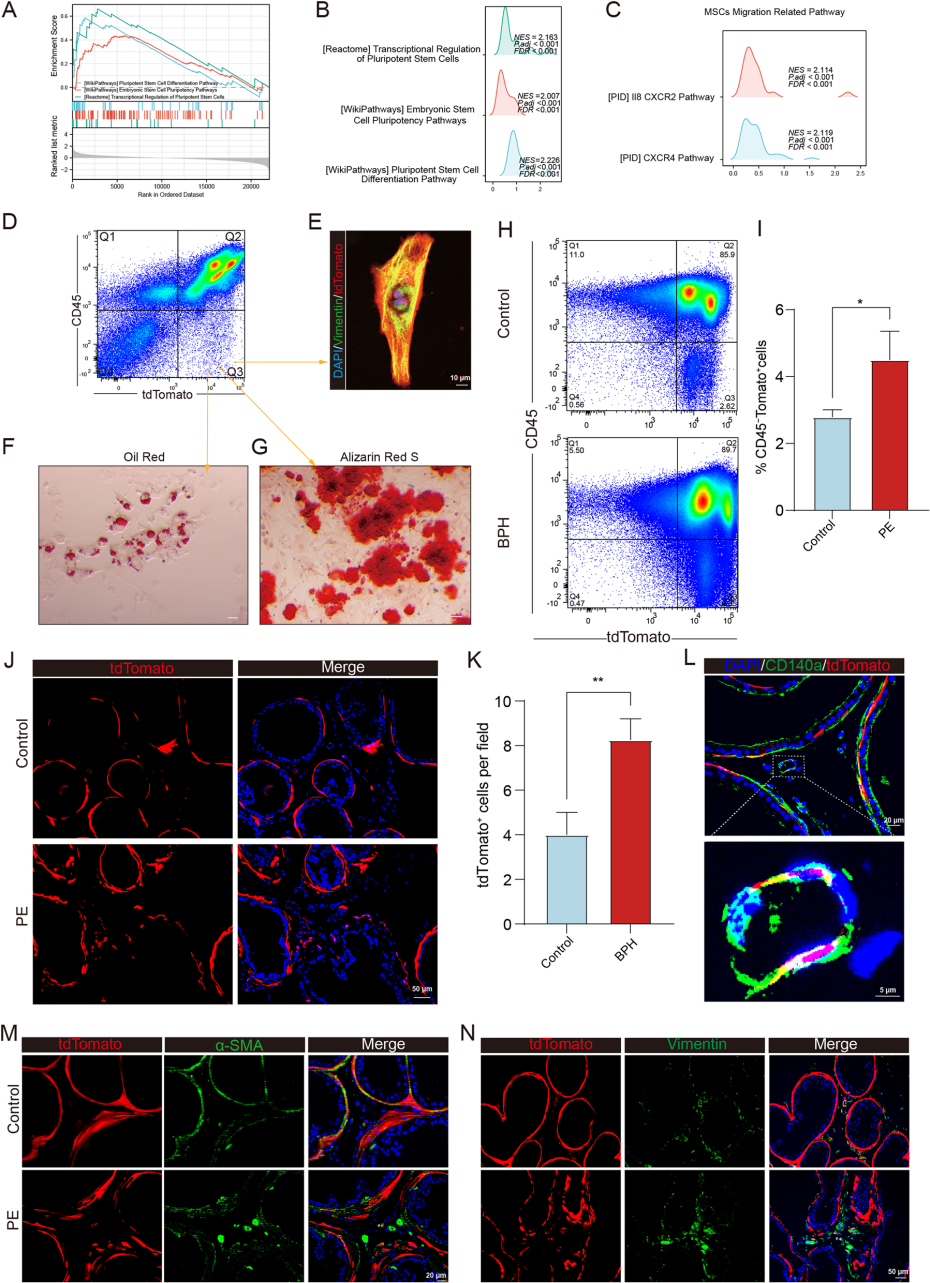
LepR^+^ cells are responsible for generating fibroblasts/myofibroblasts at the remodeling sites of the reawakening stroma. **A-B** GSEA of stem cell-related gene sets between the high and low TGF-β score groups. **C** GSEA of MSC migration related-pathway gene sets between the high and low TGF-β score groups. **D** CD45^–^ and tdTomato^+^ prostatic cells were sorted by flow cytometry for fibrogenic (**E**), lipogenic (**F**), osteogenic (**G**) induction. Scale bar = 10 μm. **H-I** Flow cytometry was used to measure the percentage of CD45^–^, and tdTomato^+^ cells in the blood of the two groups. **J-K** Immunofluorescence analysis of ventral prostate tissue sections from 12-week-old LepR-tdTomato mice treated with saline or PE. Scale bar = 50 μm. **L** Double immunofluorescence (CD140a, green; tdTomato, red) analysis of ventral prostate tissues from LepR-tdTomato mice treated with phenylephrine. Scale bar = 20 μm. **M–N** Double immunofluorescence (α-SMA or vimentin, green; tdTomato, red) analysis of ventral prostate tissues from LepR-tdTomato mice treated with PE. Scale bar = 20 or 50 μm. Data represent means ± SEMs (n = 5 per group). *p < 0.05, **p < 0.01

Victor K’s investigation had identified a population of multipotent stem cells coexisting in human prostate stromal tissue, and our previous study further demonstrated that nestin^+^ MSCs were recruited by TGF-β in prostatic stromal hyperplasia [30, 45]. To further validate the findings regarding TGF-β and MSCs, we conducted GSEA in the high TGF-β score group compared to the low TGF-β score group. As anticipated, pathway enrichment analysis revealed that the enriched genes were primarily involved in the transcriptional regulation of pluripotent stem cell pathways, embryonic stem cell pluripotency pathways, and pluripotent stem cell differentiation pathways (Fig. 3A-B). In addition, MSC migration-related pathways were significantly enriched in the high TGF-β score group (Fig. 3C).

Since LepR^+^ MSCs were identified as the main source of myofibroblasts in primary myelofibrosis, further investigations are feasible to determine whether LepR^+^ mesenchymal/stromal stem cells are recruited and differentiate into myofibroblasts or fibroblasts in prostatic stromal hyperplasia [46]. To address this issue, we crossed tdTomato^flox/flox^ mice with transgenic mice expressing an improved Cre recombinase under the control of the leptin receptor gene promoter (LepR-cre). Our PCR analysis confirmed the genotype of the LepR-tdTomato mice (Fig. S3A). Subsequent studies confirmed the multilineage differentiation potential of LepR^+^ cells, which were isolated and sorted based on their CD45-negative and tdTomato^+^ fluorescence (Fig. 3D). Their multipotency was assessed through fibrogenesis, osteogenesis, and adipogenesis assays (Fig. 3E-G).

Furthermore, LepR-tdTomato mice were utilized to investigate whether aberrant activation of TGF-β is involved in the recruitment of LepR^+^ MSCs during prostatic hyperplasia. The population of CD45^−^tdTomato^+^ cells in peripheral blood nearly doubled after PE injection compared to the control (Fig. 3H-I), and tdTomato^+^ cells also significantly increased in the prostate stroma of the PE-induced mice (Fig. 3J-K). Immunostaining of CD140a, a marker for LepR^+^ MSCs, displayed colocalization with tdTomato in the prostate stroma, as shown in Fig. 3L. Subsequent studies explored the role of LepR^+^ MSCs in prostatic stromal hyperplasia. Immunofluorescence staining of α-SMA and vimentin in prostate sections partially revealed the origin of prostate stromal cells. α-SMA staining demonstrated that smooth muscle cells were primarily derived from LepR^+^ cells (Fig. 3M), while vimentin staining also indicated that LepR^+^ cells contributed to the formation of prostatic stromal fibroblasts/myofibroblasts (Fig. 3N). These findings highlight the importance of LepR^+^ cells as an important source of stromal cells in the prostate.

###  Enhanced ROCK1 expression is crucial for the migration and differentiation of LepR^+^ MSCs

**Fig. 4:**
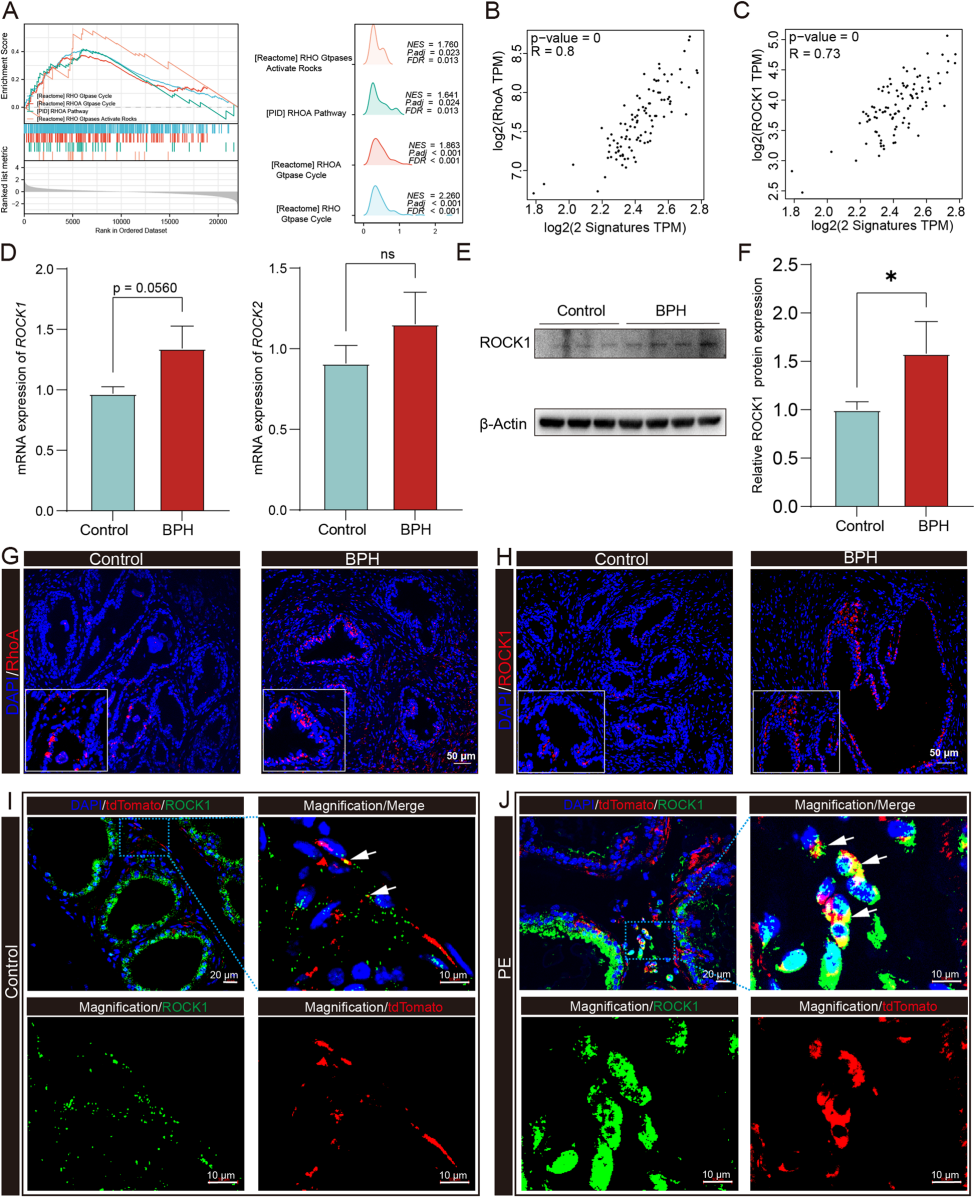
TGF-β mediates overactivated RhoA/ROCK1 signaling in BPH. **A** GSEA of RhoA pathway gene sets between high and low TGF-β score groups. **B** The Spearmen correlation of RhoA a and TGFBR1, TGFBR2 in normal prostate. **C** The Spearmen correlation of ROCK1 and TGFBR1, TGFBR2 in normal prostate. **D** ROCK1 and ROCK2 expression was determined by RT–qPCR. **E–F** Immunoblot analysis of ROCK1, in normal prostate (control) and BPH. **G** Expression of active RhoA (red) in human normal prostate(left) and BPH (right) tissues. Scale bars = 50 μm. **H** Expression of ROCK1 (red) in prostate tissues. Scale bars = 50 μm. **I-J** Double immunofluorescence (ROCK1, green; tdTomato, red) analysis of ventral prostate sections from LepR-tdTomato mice treated with or without PE. White arrows indicate double positive signals; Scale bars = 20 μm or 10 μm. Data represent means ± SDs (n = 5 per group). *p < 0.05

The molecular mechanisms of TGF-β in BPH are not fully understood, although the principal components of the canonical and noncanonical TGF-β signaling pathways have been identified. GSEA between the high and low TGF-β groups revealed that pathways enriched in the high TGF-β group included the RhoA pathway, Rho GTPase cycle and Rho GTPases activate ROCKs (Fig. 4A). In this context, we investigated the association between TGF-β and RhoA, a member of the small G-proteins belonging to the Rho GTPase family and activated by TGF-β-dependent pathways. We replicated the association between TGF-β and RhoA in GTEx prostate samples (Fig. 4B, R = 0.8). ROCK1 and ROCK2 are key downstream molecules of RhoA signaling, and the Pearson correlation coefficients between ROCK1 or ROCK2 and TGF-β receptors were 0.73 and 0.65, indicating a strong relationship (Fig. 4C, Fig. S3B).

**Fig. 5:**
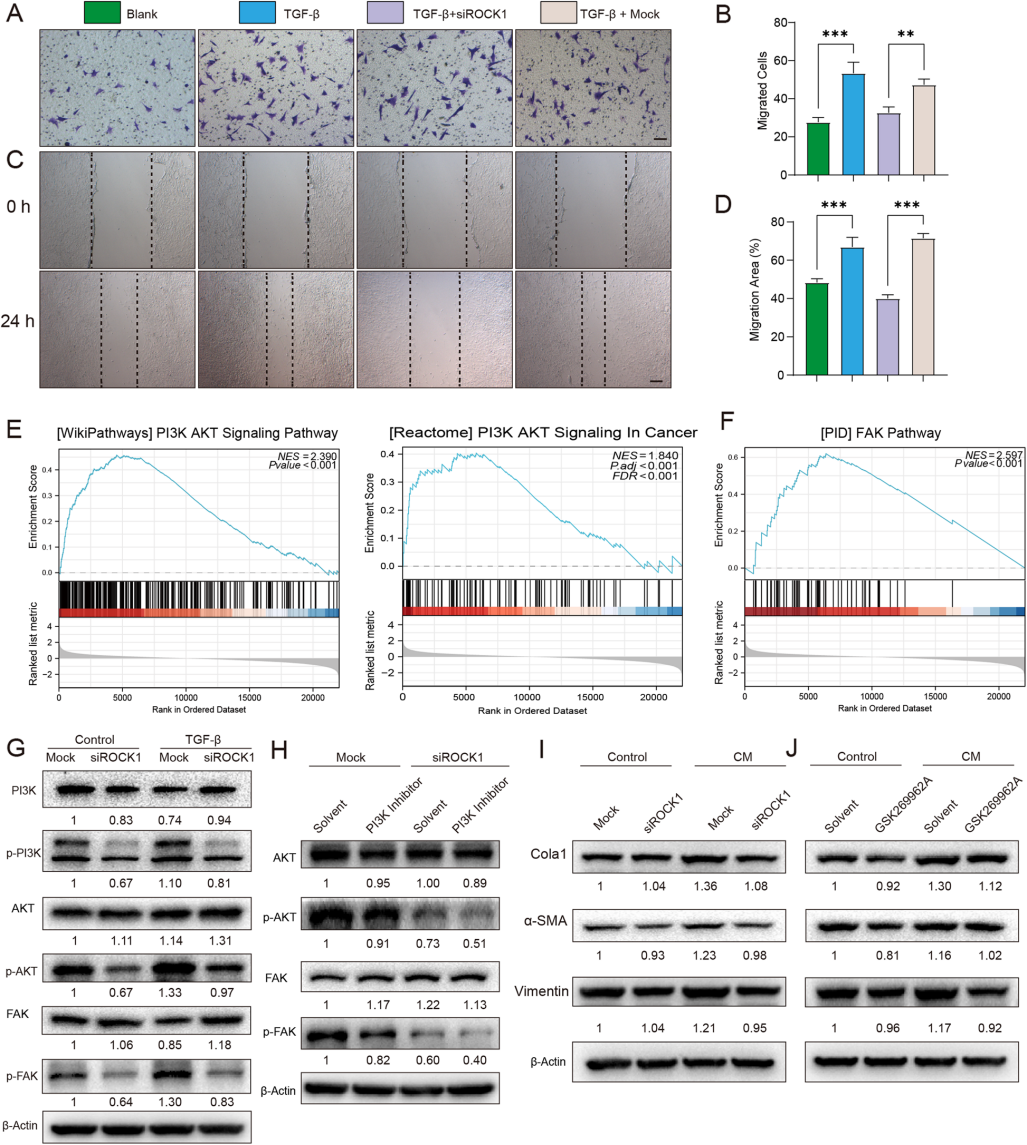
ROCK1 Regulates LepR^+^ cell mobilization and differentiation in vitro. **A-D** Transwell and wound healing assays were performed to detect LepR^+^ MSCs treated with TGF-β + siROCK1 alone or simultaneously. Scale bar = 100 μm. **E–F** GSEA of PI3K-AKT or FAK pathway gene sets between the high and low TGF-β score groups. **G-J** Immunoblot analysis of PI3K, p-PI3K, FAK, p-FAK, AKT, p-AKT, Cola1, α-SMA, vimentin in differently prepared MSC lysates (PI3K inhibitor, LY294002, 10 μM; ROCK1 inhibitor, GSK269962A, 1 μM). β-actin: loading control. Data represent the means ± SEMs (n = 3 per group). **p < 0.01, ***p < 0.001

Additionally, to further investigate the mechanism, we collected clinical specimens of normal prostate (control) and BPH and evaluated the expression levels of ROCK1 and ROCK2. RT–qPCR and immunoblotting analysis showed increased expression of ROCK1, but not ROCK2, in BPH (Fig. 4D-F). Further verification focused on ROCK1, and immunofluorescence staining confirmed enhanced expression of RhoA and ROCK1 in prostate sections from BPH patients with positive signals primarily distributed around the glands (Fig. 4G-J). The correlation analysis also revealed a strong association between ROCK1 expression and stemness of BPH tissues (Fig. S3C). Dual immunofluorescence staining demonstrated overactivation of ROCK1 in LepR^+^ MSCs within the prostate stroma of the PE-induced mice (Fig. 4I-J). This distribution of ROCK1 signals correlated with the previous finding (Fig. 3N-M) that LepR^+^ cells are also distributed around the glands. These results suggest that ROCK1 activation in LepR^+^ cells may be a critical signaling event in the TGF-β pathway in BPH. While multiple studies have demonstrated that ROCK1 plays a crucial role in cellular migration and transdifferentiation through cytoskeletal remodeling and modulation of adhesion, its role in the migration and differentiation of LepR^+^ MSCs is poorly understood. To validate the role of ROCK1, we synthesized three siRNAs targeting ROCK1. The knockdown efficiency in LepR^+^ cells was confirmed by RT–qPCR and Western blot analysis (Fig. S4A-B). In the Transwell assay, the cell count of the ROCK1 knockdown group was significantly decreased compared to the mock group after 24 h of cell inoculation (Fig. 5A-B). In the scratch wound healing assay, the cell migration ability was reduced in the ROCK knockdown group compared to the control group (Fig. 5C-D). These results demonstrate that ROCK1 is a crucial molecular regulator of the migration of LepR^+^ MSCs.

To analyze the pathways involved in LepR^+^ MSCs migration, we performed GSEA using the GSEA tool and MSigDB databases. The analysis revealed the involvement of the ‘Pluripotent Stem Cell Differentiation,’ ‘PI3K-AKT,’ and ‘FAK’ pathways (Fig. 4B and Fig. 5E-F), which have been reported to be important for the migration and differentiation of MSCs through ROCK1. Based on this finding, we hypothesized that ROCK1 and these pathways play similar roles in LepR^+^ MSCs, which we further investigated experimentally. We examined whether blocking the TGF-β-induced increase in ROCK1 through ROCK1-specific siRNA transfection into LepR^+^ MSCs changed the status of the PI3K/AKT/FAK pathway. A mock siRNA was used as a control. The p-PI3K protein level significantly decreased in the TGF-β-treated LepR^+^ MSCs following ROCK1 siRNA treatment for 48 h, whereas this decrease was not observed in the mock siRNA-treated cells (Fig. 5G). Phosphorylated of AKT (p-AKT S473) and FAK (p-FAK Tyr397), which are known to regulate cellular functions such as proliferation, apoptosis, and migration through the PI3K/AKT pathway, decreased following ROCK1 knockdown in the TGF-β-treated LepR^+^ MSCs. Furthermore, this phosphorylation was inhibited by treatment with LY294002, a broad-spectrum PI3K inhibitor (Fig. 5H).

Profibrotic mediators, including TGF-β, thrombin, and lysophosphatidic acid, act through receptors that activate ROCK1. Inhibition of ROCK1 activation could be a potent therapeutic strategy for fibrosis-related diseases. We demonstrated that silencing ROCK1 in the LepR^+^ MSCs resulted in decreased fibrosis, as evidenced by reduced expression levels of col1a1, vimentin, and α-SMA, compared to those of the mock siRNA-treated cells (Fig. 5I, Fig. S4F-H). Additionally, treatment with GSK269962A, a selective ROCK inhibitor, effectively inhibited mesenchymal differentiation, as indicated by low levels of Col1a1, vimentin, and α-SMA (Fig. 5J). These findings suggest that ROCK1 affects the migration and differentiation of LepR^+^ MSCs and could be a potential target for fibrosis-related diseases.

### Knockdown of ROCK1 signaling ameliorates prostatic stromal hyperplasia

**Fig. 6:**
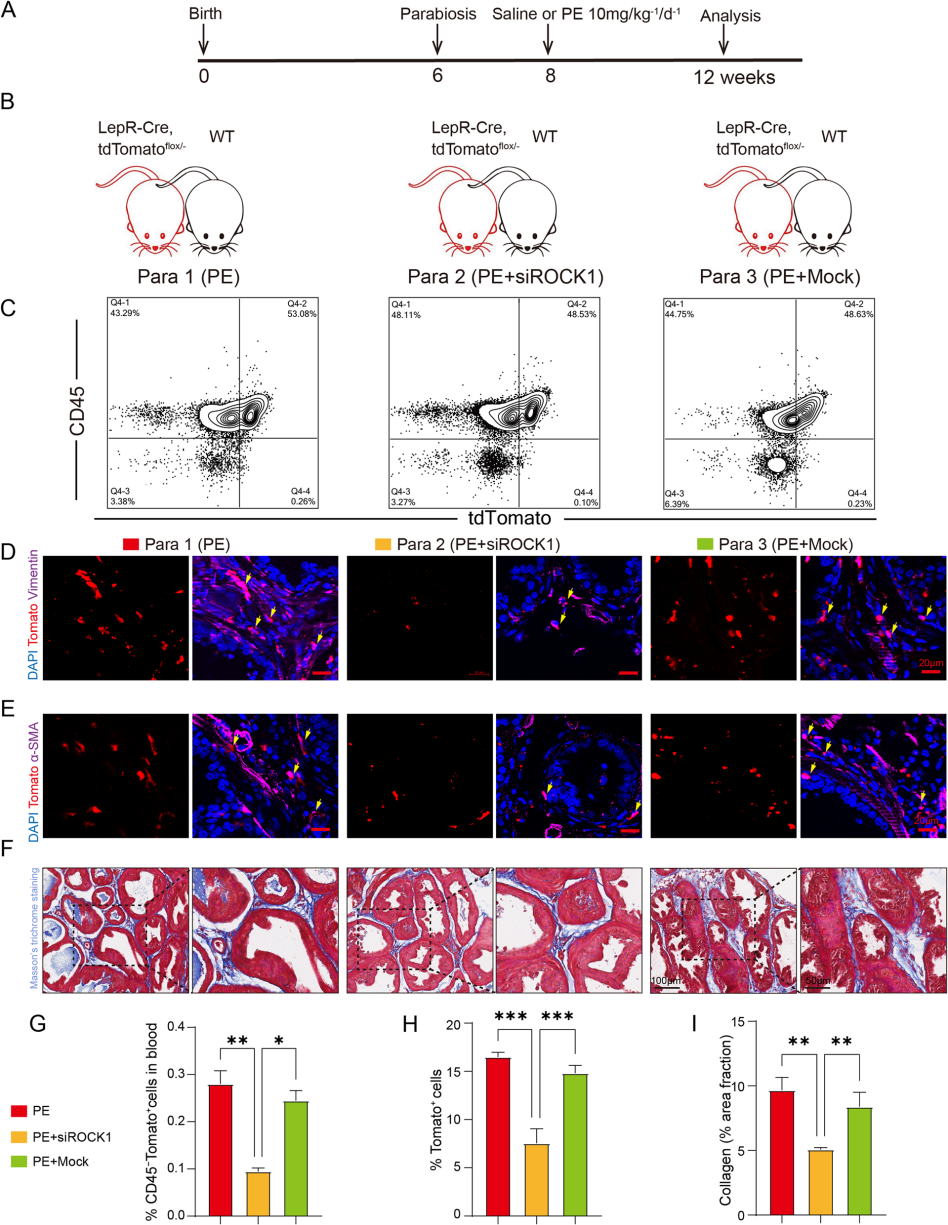
Inhibition of ROCK1 signaling inhibits LepR^+^ cells mobilization and differentiation in vivo. **A** Schematic diagram of the experimental strategy. **B** Strategies and intervention for conjoined twin mice. **C** The proportion of CD45^–^ and tdTomato^+^ cells in the peripheral blood of WT (wild-type) mice detected by flow cytometry. **D-E** Double immunofluorescence (α-SMA or vimentin, purple; tdTomato, red) analysis of ventral prostate sections from WT mice treated with siROCK1 or Mock. **F** Representative images of Masson trichrome staining of collagen deposition in WT mice ventral prostate sections. Scale bars = 20 μm. **G** Statistical analysis of flow cytometry detection. **H** Statistical analysis of tdTomato^+^ cells in WT mice prostate tissues. **I** Statistical analysis of collagen deposition in WT mice ventral prostate sections. Data represent the means ± SDs (n = 3 per group). *p < 0.05, **p < 0.01, ***p < 0.001

To validate the role of ROCK1 in the mobilization and differentiation of LepR^+^ MSCs and prostate stromal formation in vivo, we conducted heterochronic parabiosis experiments. We joined the vasculature of Lepr-tdTomato mice (carrying the tdTomato marker in LepR^+^ cells) with wild-type mice (Fig. 6A-B). All parabiotic pairs were treated with PE, a compound used to induce prostatic hyperplasia. We injected siRNAs targeting ROCK1 or an equal negative control siRNA (NC) into the cojoined wild-type mice.

Flow cytometry analysis of peripheral blood revealed that treatment with ROCK1 siRNA reduced the proportion of tdTomato^+^ CD45^−^ cells in the circulation (Fig. 6C and G). Furthermore, treatment with ROCK1 siRNA dramatically decreased the presence of tdTomato^+^–labeled α-SMA^+^ or vimentin^+^ fibroblast/myofibroblast cells and collagen deposition in the prostate stromal tissue of PE-treated cojoined wild-type mice compared to the mice treated with the NC siRNA (Fig. 6D-F, H- I). These in vivo and in vitro ROCK1 knockdown data confirmed that reduced ROCK1 signaling inhibits the recruitment and differentiation of LepR^+^ cells in prostatic hyperplasia. The results provide further evidence for the critical role of ROCK1 in the migration and differentiation of LepR^+^ MSCs and its involvement in the formation of prostate stromal tissue.

### Potential therapeutic options for hyperactivation of ROCK1 signaling in BPH

**Fig. 7:**
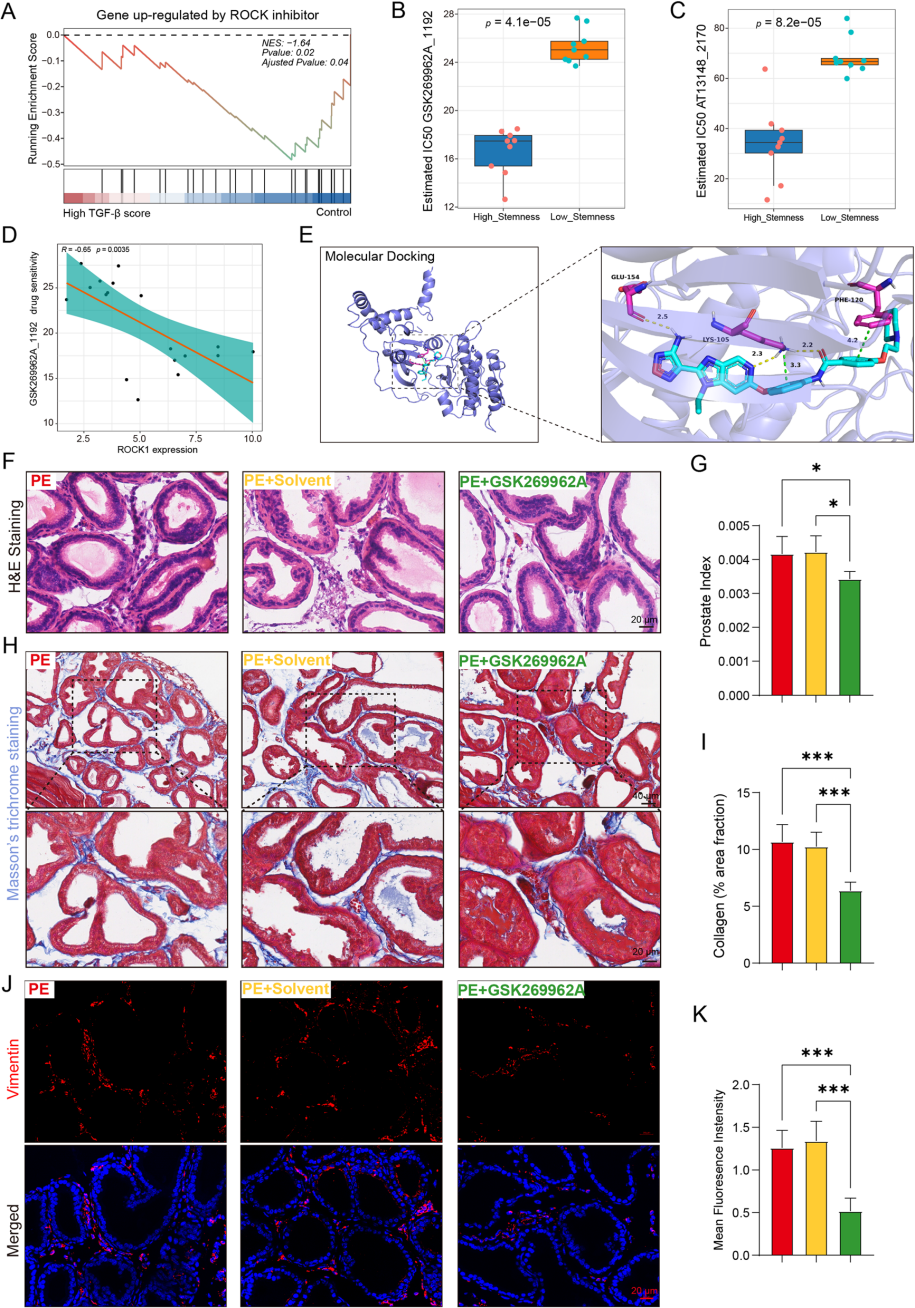
GSK269962A targeting ROCK1 attenuates prostatic stromal hyperplasia. **A** GSEA of ROCK-related signatures in the high TGF-β score group, showing that genes upregulated by ROCK inhibitors are negatively enriched compared to those of the control samples. **B-C** ROCK1 inhibitor prediction of the high stemness groups. **D** Correlation analysis of the IC50 of GSK269962A and ROCK1 expression. **E** Molecular docking simulation of GSK269962A and ROCK1. **F** HE staining of ventral prostate sections from PE-, PE + solvent- or PE + GSK269962A treated mice. Scale bars = 20 μm. **G** Prostate index of mice treated with PE, PE + solvent or PE + GSK269962A. **H-I** Representative images of Masson trichrome staining of collagen deposition on ventral prostate sections and statistical analysis. Pictures were taken at two magnifications: × 200 (upper panels) and × 400 (lower panels). Scale bars = 20 μm. **J-K** Vimentin immunofluorescence staining and statistical analysis. Scale bars = 20 μm. Data represent the means ± SEMs (n = 5 per group). *p < 0.05, ***p < 0.001

To further elucidate the therapeutic potential of ROCK inhibitors in TGF-β-overactive BPH, we examined the enrichment of ROCK1 signaling pathways using GSEA. We found a significant negative enrichment of genes upregulated by the ROCK1 inhibitor in normal prostate tissue (Fig. 7A). This finding suggests that ROCK1 inhibitors might counteract the overactive ROCK1 signaling observed in BPH. Then, we evaluated and observed the differences in sensitivity to 198 common drugs in BPH patients between the two groups. To further screen the ROCK1 inhibitors for prostatic hyperplasia, drug sensitivities were predicted and differences in drug sensitivities between the high- and low-stemness samples were analyzed. The results showed that patients in the high stemness group were more sensitive to the ROCK1 inhibitors GSK269962A and AT1314, implying that these drugs have better therapeutic effects in the overactivation of TGF-β/ROCK1 in BPH. (Fig. 7B, C). Additionally, we specifically focused on GSK2966692A, a known ROCK inhibitor with anti-fibrotic properties. Correlation analysis showed that the IC50 was negatively correlated with the expression of ROCK1 (Fig. 7D). In silico studies and molecular docking revealed the formation of multiple hydrogen bonds between GSK2966692A and key residues of ROCK1, such as Glu154 and LYS105. The binding energy of the protein–ligand complex was -10.1 kcal/mol, indicating a strong interaction (Fig. 7E). These results provide a good reference for clinical medication.

To further validate the therapeutic efficacy of GSK2966692A, we conducted animal studies. Histological examination using HE and Masson’s staining demonstrated a reduction in the interstitial component and collagen deposition in the prostates of the mice treated with GSK2966692A. This reduction was consistent with the decreased prostate index observed in the treated group (Fig. 7F-I). Immunofluorescence analysis of prostate sections from the GSK2966692A-treated mice confirmed a significant decrease in vimentin^+^ fibroblasts/myofibroblasts cells compared to those of the group treated with the solvent (Fig. 7J-K). Overall, our combined analyses and experimental results indicate that GSK2966692A is a potential drug for the treatment of prostatic stromal hyperplasia in BPH. Its ability to inhibit ROCK1 signaling and reduce fibrosis-related features suggests that this drug could be a valuable therapeutic option for TGF-β/ROCK1-overactive BPH.

## Discussion

The theory of embryonic reawakening, proposed by McNeal in 1976, has become an important concept in the pathogenesis of BPH [11, 47]. However, debate is still ongoing, and direct evidence linking embryonic reawakening to the occurrence of BPH is lacking [7]. The initial theory suggests that restricted growth in the distal transition zone plays a significant role in embryonic reawakening in BPH [14]. Recent advancements in immunohistochemical techniques, lineage tracing, and flow cytometry have revealed the presence of multipotent stem cells and stem cell-like stromal cells in the transition zone of BPH. Lin et al. reported that primary stromal cells from BPH patients possess MSC markers, strong proliferative potential, and the ability to differentiate or transdifferentiate into myogenic, adipogenic, and osteogenic lineages. Brennen et al. showed that MSCs represent 0.01–1.1% of the total cells present in core biopsies of primary human prostatectomies. MSCs in these prostatectomy samples are positive for FAP, CD90, CD73, and CD105 and negative for hematopoietic lineage markers [48]. Therefore, Brennen's hypothesis innovatively proposes infiltrating MSCs as the driver of occurrence in BPH [14]. Previous study clarified that the primary reservoir of LepR^+^ cells is in the adult bone marrow and confirmed the involvement of LepR^+^ MSCs in embryonic and postnatal bone growth [49, 50]. The recruitment mechanism of LepR^+^ cells is currently unclear, but there are reports in the literature that PDGFα, IGF-1, MCP-1, and CXCL12/CXCR4 are involved in the recruitment of MSCs; whether the recruitment of LepR + cells involve these molecules requires further study [51]. In fact, active TGF-β released from inflammatory or injured tissues recruits MSCs for tissue repair/remodeling. The continuous exposure of transition zone tissue to urinary components and an inflammatory microenvironment generated by self-antigens can recruit bone marrow-derived MSCs and induce stromal proliferation through paracrine signaling, leading to nodular growth of the prostate. TGF-β also emerged as an important cytokine recruiting MSCs, inducing prostate stromal hyperplasia [14, 52]. In this study, we demonstrated significant activation of the TGF-β/ROCK1 pathway, which is associated with embryonic development, in BPH tissues. For the first time, we quantified the stemness of BPH tissues using ssGSEA and found a strong positive correlation between the stemness of BPH tissues and the TGF-β pathway score, revealing that BPH tissues with high TGF-β scores may have a greater degree of stemness, as they can recruit multipotential MSCs to the prostate, providing direct validation of the relationship between BPH tissue stemness and TGF-β. This finding provides robust evidence supporting the theory of embryonic reawakening in the pathogenic mechanisms of BPH.

The TGF-β superfamily is known to regulate various cellular functions, including EMT, fibrosis, cellular immunity, proliferation, apoptosis, and dedifferentiation. The correlation analysis in this study confirmed this positive relationship between TGF-β and EMT and fibrosis (Fig. S2C). The immunoscore represents the level of immune cell infiltration in the tissue, but more research is needed to understand the relationship between the TGF-β pathway score and immunoscore in BPH (Fig. S2D) Excessive activation of the TGF-β pathway has been identified as an important molecular driver in BPH. Targeting excessive activation of TGF-β could serve as a potential therapeutic strategy for BPH. Several TGF-β inhibitors are currently undergoing clinical trials or are in clinical use for various diseases. For instance, fresolimumab, a human anti-TGF-β monoclonal antibody, has demonstrated efficacy against melanoma and renal cell activity in clinical trials, while pirfenidone can directly inhibit TGF-β production and alleviate pulmonary fibrosis [53, 54]. Investigating changes in prostate volume and IPSS scores in these subjects may yield novel insights into BPH treatment. The imbalance in androgen and estrogen levels with increasing age in prostate tissue also contributes to BPH progression. Elevated levels of estrogen in the prostate tissue promote hyperplastic growth and abnormal tissue remodeling in BPH [55–57]. Interestingly, the profibrotic effects of TGF-β are similar to those of estrogen, and bioinformatic analysis showed a strong positive correlation between the TGF-β pathway score and the estrogen receptor (ER) score, while the androgen receptor (AR) score did not appear to be associated with TGF-β (Fig. S2E-F). Further research is needed to explore this association in more detail.

Although we have shown through bioinformatic analysis, lineage tracing, and a parabiosis mouse model that aberrant TGF-β/ROCK1 signaling correlates to some extent with prostate stemness, the association was confirmed only in prostate stroma and not in epithelial tissues. Many recent studies have found that epithelial stem cells play an essential role in the development, progression, and therapeutic resistance of BPH [13, 58]. Wang et al. also identified Zeb1^+^ prostate epithelial cells, which are multipotent prostate basal stem cells capable of self-renewal and generating functional prostatic glandular structures at the single-cell level [59]. Using single-cell sequencing technology, the Strand lab identified a new type of epithelial cell from the donor's prostate, namely "club" cells [60]. Further studies clarified the stem-like and castration-insensitive properties of these cells [61]. SCGB1A1 and SCGB3A1 are considered characteristic marker genes of club cells. Interestingly, the expression of both characteristic marker genes increased in high TGF-β samples, an observation worthy of further investigation (Fig. S3D).

The enlargement of the transition zone volume is an important characteristic of BPH progression [1]. This study found a positive correlation between the stemness index represented by multiple stemness gene sets and the transition zone volume (although only the Hs_SC_Shats gene set showed a significant positive correlation) (Fig. S2B). This finding is consistent with the report by Yan et al., indicating higher expression of stemness genes in the transition zone of the prostate [38]. In this study, the stemness index of most gene sets showed a negative correlation with the immunoscore, although only two correlations were statistically significant (Fig. S3E, red font). This observation suggests that BPH with higher stemness may exhibit lower levels of immune cell infiltration, which seems contradictory to the recruitment of MSCs in an inflammatory microenvironment.

Accumulating evidence suggests a crucial role for the ROCKs system in the pathogenesis of various urogenital disorders [62]. ROCKs, as serine/threonine kinases, are essential contributors to the regulation of the cytoskeleton [63]. Prostate fibrosis, characterized by the replacement of normal tissue with noncompliant fibrotic tissue, is associated with ROCK-mediated processes [64]. While ROCK1 appears more crucial in mediating fibrosis, ROCK2 also contributes to this phenomenon [65]. In addition, ROCK1-mediated EMT in prostatic epithelium exacerbates the accumulation of mesenchymal-like cells [66]. The role of ROCK in MSCs remains largely unknown, although studies suggest that ROCK inhibitors, such as Fasudil and Y27632, influence MSC differentiation [25, 67]. This research confirmed the role of ROCK1 signaling in regulating the migration and differentiation of MSCs, as well as its involvement in remodeling the prostate stroma. ROCK1 mediates MSC migration, at least in part, through the PI3K/AKT/FAK pathway.

Considering the implication of the ROCK molecule in BPH, its inhibitors present potential therapeutic agents. GSK269962A, a ROCK inhibitor, has demonstrated efficacy in alleviating cyclophosphamide-induced detrusor overactivity and bladder inflammation [68, 69]. Our research affirms the effectiveness of the small molecule inhibitor GSK269962A in mitigating interstitial prostatic hyperplasia. Pan et al. conducted analyses on potential cytotoxic side effects of GSK269962A in C57BL/6 mice, revealing no overt harmful effects on vital organs or the hematological system, and other study suggest that some side effects of GSK269962A were hypotension and cardiovascular collapse [70, 71]. However, we cannot quantify the side effects of GSK269962A due to the limited experimental conditions.

In summary, this study provides evidence supporting the theory of embryonic reawakening in BPH. The results demonstrate a positive correlation between the TGF-β pathway score, stromal score, and stemness index in BPH tissues. The study also highlights the role of TGF-β in EMT, its potential interaction with estrogen signaling, and its influence on immune homeostasis. Furthermore, we elucidate the involvement of ROCK1 signaling in BPH pathogenesis and suggest the potential of the ROCK1 inhibitor GSK269962A as a therapeutic option.

### Supplementary Information

Supplementary Material 1.

Supplementary Material 2.

## Data Availability

No datasets were generated or analysed during the current study.

## Abbreviations

TGF-β: Transforming growth factor-β
ROCK1: Rho-associated protein kinase 1
BPH: Benign prostatic hyperplasia
LepR: Leptin receptor
LUTS: Lower urinary tract symptoms
MSCs: Mesenchymal stem cells
EMT: Epithelial-mesenchymal transition
PE: Phenylephrine
HE: Hematoxylin and Eosin
OCT: Optimal cutting temperature compound
IOD: Integral optical density
BSA: Bovine serum albumin
PBS: Phosphate-buffered saline
RT–qPCR: Real‐time quantitative PCR
GAPDH: Glyceraldehyde-3-phosphate dehydrogenase
PS: Penicillin-streptomycin
ssGSEA: single-sample gene set enrichment analysis
GEO: Gene expression omnibus
GTEx: Genotype-tissue expression
ROCK2: Rho-associated protein kinase 2
α-SMA: Alpha-smooth muscle actin
siRNA: Small interfering RNA
Glu: Glutamic acid
LYS: Lysine
FAP: Fibroblast activation protein
SCGB1A1: Secretoglobin family 1A member 1
SCGB3A1: Secretoglobin family 3A member 1
