# Supplementary material for: Aberrant activation of TGF-β/ROCK1 enhances stemness during prostatic stromal hyperplasia
Source: Cell Commun Signal. 2024 May 6;22:257. doi: 10.1186/s12964-024-01644-4 (PMC11071275; doi:10.1186/s12964-024-01644-4)
Supplement: Supplementary file 1 — Supplementary Material 1. [file 12964_2024_1644_MOESM1_ESM.pdf]

Figure S1 TGF-β signaling is activated during prostatic stromal hyperplasia in PE-induced mice

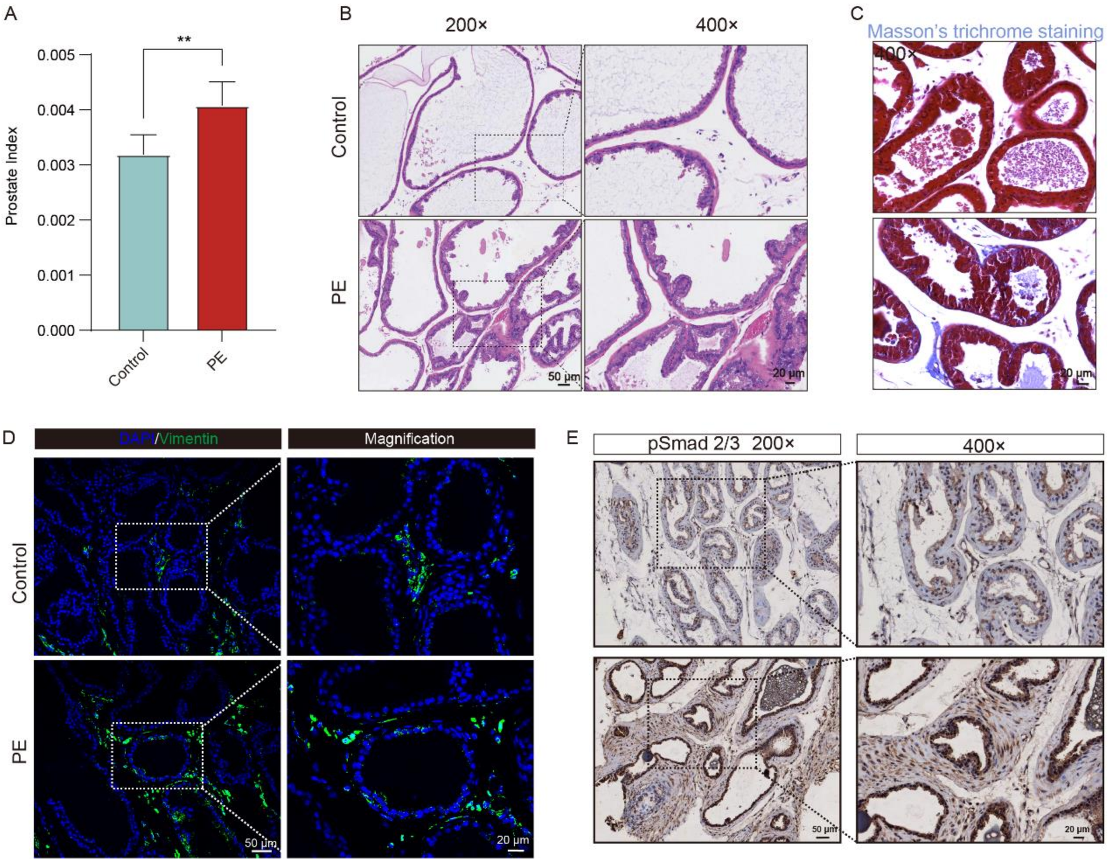

(A)Prostate index of saline (control) or PE (BPH) treated mice.

(B) HE staining of the ventral lobe prostate of the control or BPH mice. Scale bars = 50 μm.

(C) Representative images of Masson trichrome staining of collagen deposition in control or PE treated mice ventral prostate tissues. Scale bars = 20 μm.

(D) Vimentin (green) immunofluorescence staining. Scale bars = 20 μm.

(E) Expression level of p-Smad2/3 protein in control or PE treated mice ventral prostate tissues. Scale bars = 20 μm.

Figure S2 Stemness index and TGF-β score validation and correlation analysis.

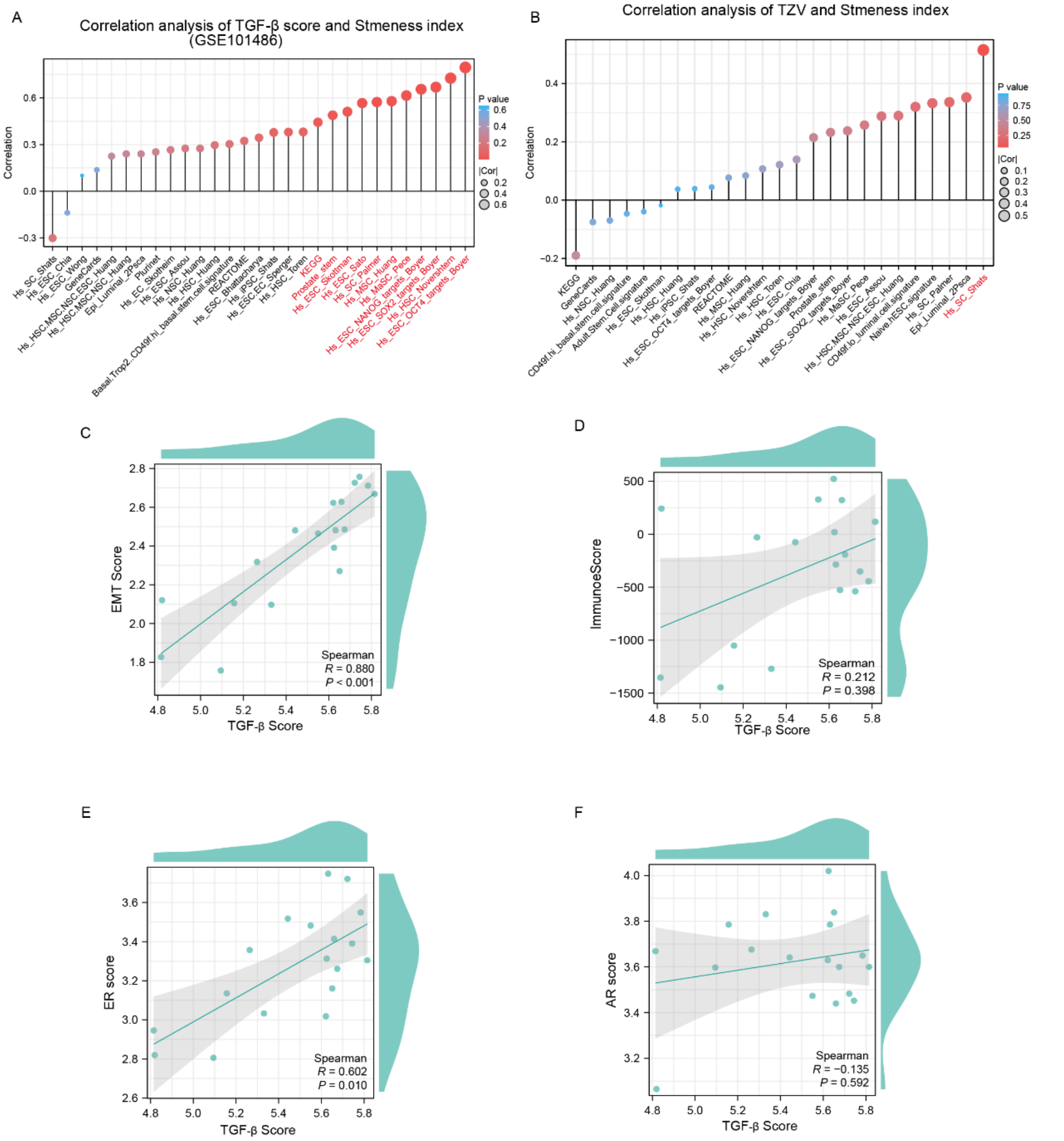

(A) Validation of the correlation between the TGF-β score and stemness index in GSE101486.

(B) Spearman analysis of the correlation between the stemness index and transition zone volume (TZV). This correlation is statistically significant (red font).

(C-F) Spearman analysis of the correlation between TGF-β score and EMT score (C), immunoscore (D), ER score (E), and AR score (F).

Figure S3 Correlation analysis and mouse genotyping.

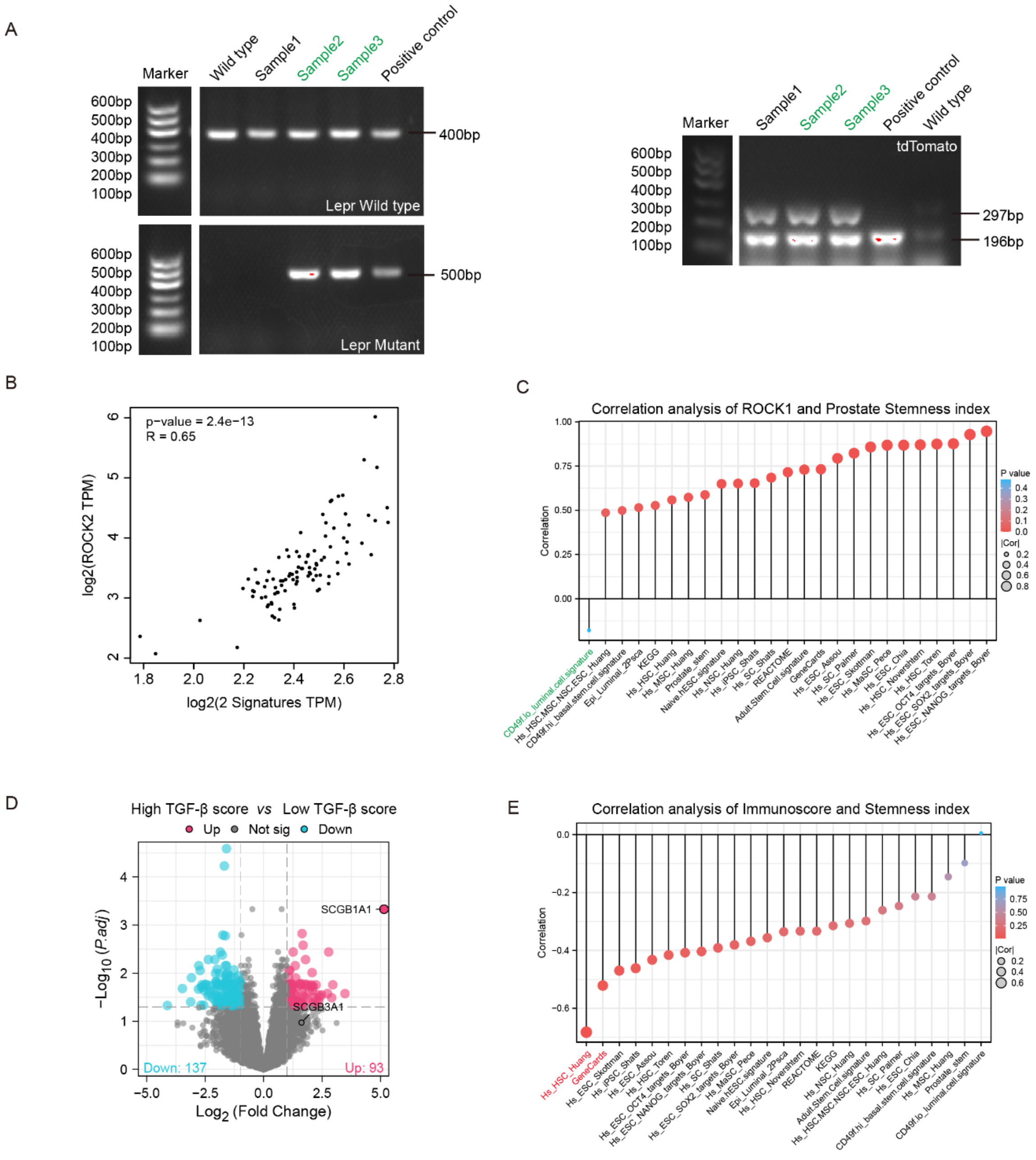

(A) LepR-tdTomato were identified by the PCR of the genomic DNA derived from tail using primers as described in TableS1. The target mice were presented in green font.

(B) Spearman analysis of the correlation between ROCK2 and TGF-β receptors.

(C) Spearman analysis of the correlation between ROCK1 and the prostate stemness index. This correlation is statistically significant, except for the green font.

(D) Volcanic map illustrating the differentially expressed mRNA between the high and low TGF-β

score groups.

(E) Spearman analysis of the correlation between the stemness index and immunoscore. This correlation is statistically significant (red font).

Figure S4 The siRNA screen suggested that ROCK1 could suppress MSC fibrogenesis.

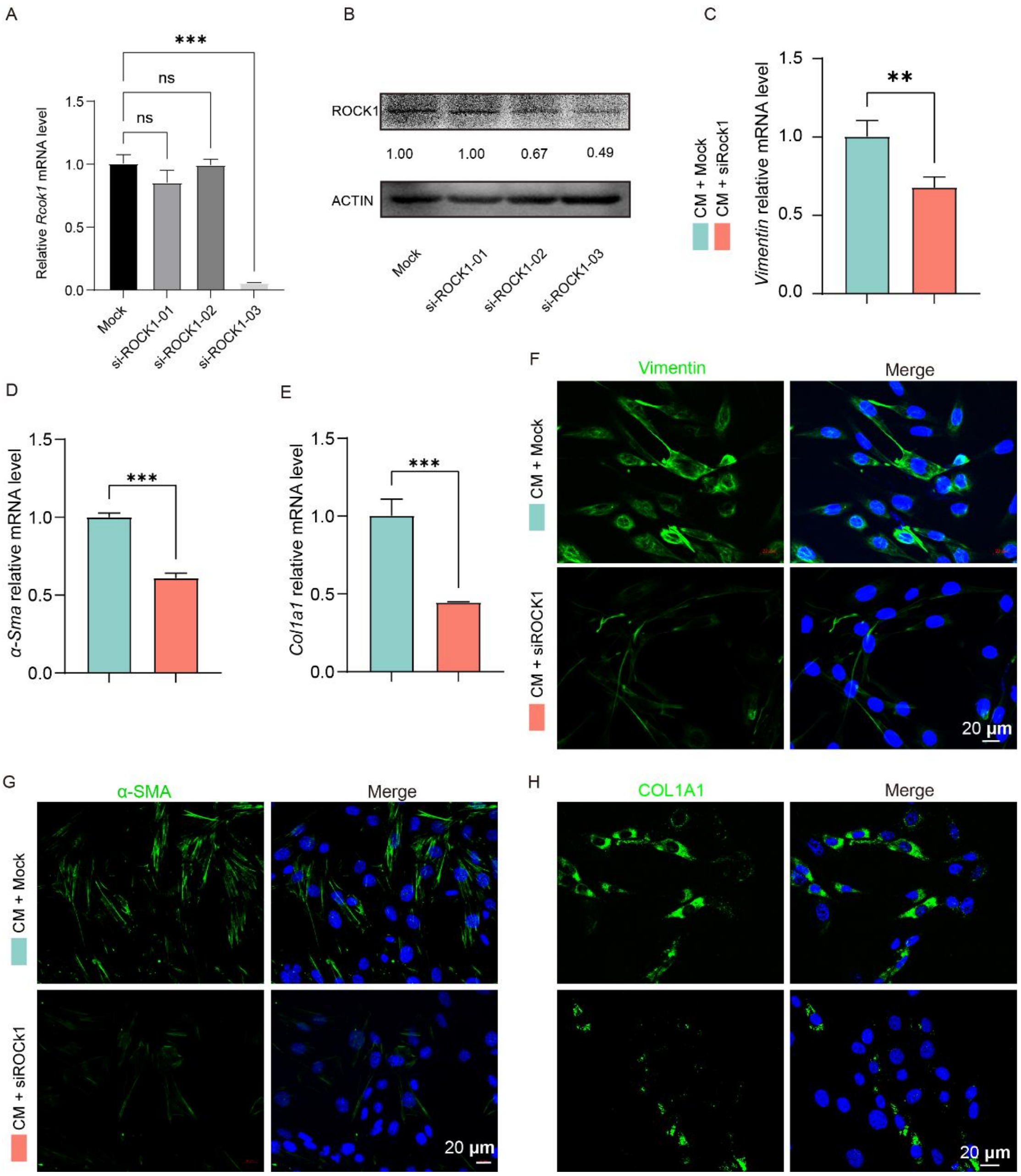

(A-C) The silencing efficiencies were assessed by RT–qPCR and Western blotting.

(D-F) Vimentin, α-SMA, and COL1A1 were measured by RT-qPCR and immunofluorescence.

Scale bars = 20 μm. Data represent means ± SEMs (n=5 per group). $^{**}p < 0.01$, $^{***}p < 0.001$.
